# Health Electronic Assessment of Risks and Trends using Biometric Equipment and Technology (HEARTBEAT): study design and rationale

**DOI:** 10.1093/ehjdh/ztag132

**Published:** 2026-08-21

**Authors:** Nassir Marrouche, Christian Massad, Han Feng, Ala Assaf, Mayana Bsoul, Yara Menassa, Ghassan Bidaoui, Qussay Marashly, Chanho Lim

**Affiliations:** Tulane Research Innovation for Arrhythmia Discovery, Tulane University School of Medicine, 1430 Tulane Ave, New Orleans, LA 70112, USA; Tulane Heart and Vascular Institute, Tulane University School of Medicine, 1430 Tulane Ave, New Orleans, LA 70112, USA; Tulane Research Innovation for Arrhythmia Discovery, Tulane University School of Medicine, 1430 Tulane Ave, New Orleans, LA 70112, USA; Tulane Heart and Vascular Institute, Tulane University School of Medicine, 1430 Tulane Ave, New Orleans, LA 70112, USA; Tulane Research Innovation for Arrhythmia Discovery, Tulane University School of Medicine, 1430 Tulane Ave, New Orleans, LA 70112, USA; Tulane Heart and Vascular Institute, Tulane University School of Medicine, 1430 Tulane Ave, New Orleans, LA 70112, USA; Tulane Research Innovation for Arrhythmia Discovery, Tulane University School of Medicine, 1430 Tulane Ave, New Orleans, LA 70112, USA; Tulane Heart and Vascular Institute, Tulane University School of Medicine, 1430 Tulane Ave, New Orleans, LA 70112, USA; Tulane Research Innovation for Arrhythmia Discovery, Tulane University School of Medicine, 1430 Tulane Ave, New Orleans, LA 70112, USA; Tulane Heart and Vascular Institute, Tulane University School of Medicine, 1430 Tulane Ave, New Orleans, LA 70112, USA; Tulane Research Innovation for Arrhythmia Discovery, Tulane University School of Medicine, 1430 Tulane Ave, New Orleans, LA 70112, USA; Tulane Heart and Vascular Institute, Tulane University School of Medicine, 1430 Tulane Ave, New Orleans, LA 70112, USA; Tulane Research Innovation for Arrhythmia Discovery, Tulane University School of Medicine, 1430 Tulane Ave, New Orleans, LA 70112, USA; Tulane Heart and Vascular Institute, Tulane University School of Medicine, 1430 Tulane Ave, New Orleans, LA 70112, USA; Tulane Research Innovation for Arrhythmia Discovery, Tulane University School of Medicine, 1430 Tulane Ave, New Orleans, LA 70112, USA; Tulane Heart and Vascular Institute, Tulane University School of Medicine, 1430 Tulane Ave, New Orleans, LA 70112, USA; Tulane Research Innovation for Arrhythmia Discovery, Tulane University School of Medicine, 1430 Tulane Ave, New Orleans, LA 70112, USA; Tulane Heart and Vascular Institute, Tulane University School of Medicine, 1430 Tulane Ave, New Orleans, LA 70112, USA

**Keywords:** Digital health, Wearable technologies, Cardiovascular disease, Biosensors, Detection, Prevention

## Abstract

**Aims:**

Cardiovascular disease remains the leading driver of morbidity and mortality worldwide. As consumer smartwatches become ubiquitous, they offer a practical platform for continuous, real-world phenotyping, capturing passive and active biometrics that may detect occult disease and anticipate adverse cardiovascular events. Scaled deployment of these devices could enable remote screening, longitudinal monitoring, and earlier risk mitigation beyond traditional episodic care.

**Methods and results:**

The Health Electronic Assessment of Risks and Trends using Biometric Equipment and Technology (HEARTBEAT) study is a prospective, single-arm wearable study that has enrolled 897 participants since 10 October 2024. The primary objectives are to: (1) define associations between smartwatch-derived biometrics and incident cardiovascular events; (2) derive biometric profiles that support identification of underlying cardiometabolic and cardiovascular conditions; and (3) apply artificial intelligence to improve prediction of cardiovascular events. Participants are screened, consented, and enrolled in person or remotely. Data are captured through electronic medical record (EMR) extraction and a companion smartphone application. Participants are followed for 12 months, with outcomes adjudicated on an ongoing basis using EMR review and app-based surveys.

**Conclusion:**

The HEARTBEAT study will test whether scalable, consumer-grade wearables can move from wellness tracking to clinically meaningful signal, identifying comorbidities and predicting adverse cardiovascular outcomes in routine care settings. If successful, the study will help establish an evidence base for smartwatches as validated digital health tools for remote screening and continuous cardiovascular monitoring.

## Graphical Abstract

**Graphical Abstract:**
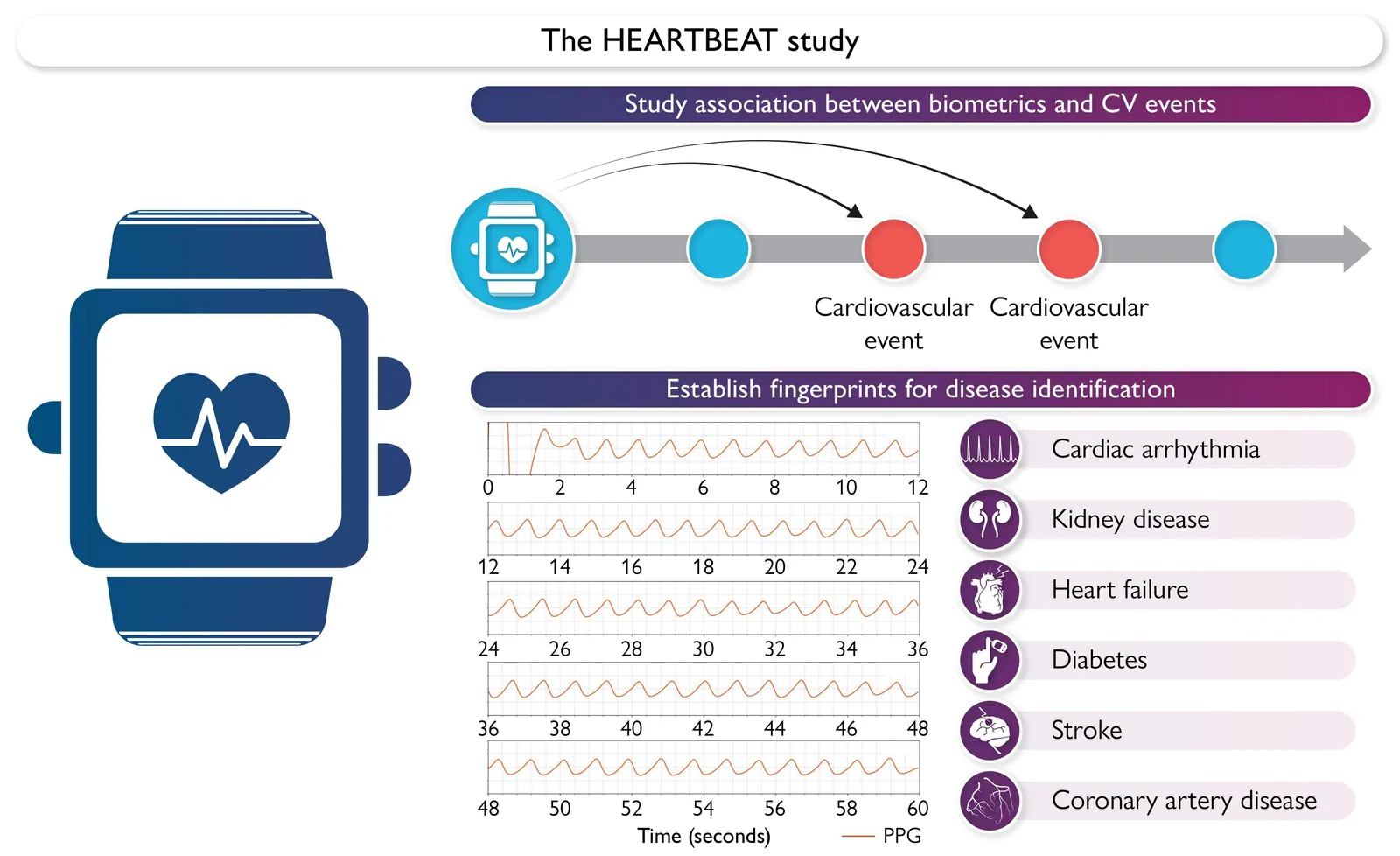
HEARTBEAT Study’s Aims. Schematic overview of the study aims. The wearable device is shown on the left, and the study objectives are depicted on the right. Study Aim 1 examines associations between wearable-derived biometric data and cardiovascular events. Study Aim 2 seeks to establish biometric fingerprints for the identification of cardiovascular and cardiometabolic diseases, including arrhythmias, kidney disease, heart failure, diabetes, stroke, and coronary artery disease.

## Introduction

Cardiovascular diseases (CVDs) are the leading cause of mortality worldwide, responsible for approximately 32% of all global deaths in 2019, with heart attacks and strokes accounting for 85% of these deaths.^1^ CVDs also impose morbidity, reduce quality of life, and increase healthcare costs.^2,3^

Despite prevention efforts, Mohebi *et al*.^4^ project a major increase in US CVD through 2060, including an increase in diabetes, hypertension, dyslipidaemia, and obesity by 15.4, 34.7, 27.1, and 19.4 million, respectively. This is paralleled by an expected increase in the prevalence of ischaemic heart disease, heart failure (HF), myocardial infarction and stroke. These projections also expose racial and ethnic disparities with CVD risk factors expected to rise disproportionately among minority populations (Hispanic, non-Hispanic Black, and other non-Hispanic), while declining among White populations.^4^

Addressing this trajectory requires proactive prevention and equitable care,^5^ areas where technological innovation has demonstrated impact. Rhythm monitoring exemplifies this impact, from 24- to 48-h Holter monitors,^6^ to extended patch-based monitors such as the Zio Patch®,^7^ and now to FDA-cleared consumer wearables like the Samsung Galaxy Watch^8^ and Apple Watch,^9^ alongside smartphone-based electrocardiography (ECG) devices such as the Kardia Mobile.^10^ These innovations have ushered in a new era of decentralized, continuous, and convenient cardiac monitoring.

Emerging evidence reflects this paradigm shift. One trial comparing conventional ECG monitoring to a remote single-lead ECG device paired with a smartphone application demonstrated a significant reduction in healthcare visits during the post-ablation period.^11^ The MIRACLE-AF trial further showed that integrating telemedicine into atrial fibrillation (AF) care improves both adherence to treatment and cardiovascular outcomes.^12^

We are now at a critical juncture in cardiovascular medicine. In an era increasingly defined by individualized care, the next major advance in management is poised to be digital. Harnessed effectively, digital health tools offer the potential to deliver personalized insights, empower participants to take ownership of their cardiovascular health, and provide clinicians with real-time data on patient status and therapeutic response.

## Rationale for the HEARTBEAT study

Wearable technologies and artificial intelligence (AI) have made personalized cardiovascular health increasingly attainable. Traditional prevention relies on risk estimates, such as the 10-year atherosclerotic cardiovascular disease (ASCVD) risk score, to guide treatment decisions,^13^ limited by its dependence on static variables like age, sex, race, and single-point clinical measurements. In contrast, wearable devices, which are compact body-worn technologies equipped with sensors such as accelerometers, photoplethysmography (PPG), and ECG, provide real-time, longitudinal insights,^14,15^ supporting a shift from static risk estimation toward individualized, proactive cardiovascular care.

The feasibility of clinical applications for these sensors has been shown across a growing number of applications for detecting AF.^16,17^ Beyond arrhythmia detection, advances in signal processing and machine learning have enabled the use of wearable devices to estimate other critical metrics like blood glucose and blood pressure.^18,19^ Both have been achieved by utilizing features of the PPG wave to enable convenient and continuous patient-centred monitoring. Importantly, wearable biometric features have also been shown to correlate clinically; for instance, temporal changes in resting heart rate (HR) have been linked to HF and ischaemic heart disease.^20,21^

The volume and complexity of wearable data create a need for AI-based analysis. AI is already established across echocardiography, nuclear cardiology, ECG, and primary prevention.^22,23^ Machine learning models have demonstrated superior risk stratification even beyond traditional ASCVD variables by modelling complex, nonlinear relationships.^24,25^ Wearables augmented by AI may therefore support lifestyle modification, prevention, and reductions in the $730 billion in annual US healthcare costs attributable to modifiable CVD risk factors.^26^ The question is: Can a unified, multipurpose sensing platform be developed to address the heterogeneous demands of modern healthcare?

The Health Electronic Assessment of Risks and Trends using Biometric Equipment and Technology (HEARTBEAT) study addresses this question by integrating wearable technology, AI, and CVD research. HEARTBEAT leverages multimodal data from commercial wearables to study the interconnected spectrum of CVDs. Enrolment focuses on participants with at least one established cardiovascular diagnosis, including AF, HF, and coronary artery disease (CAD), enriching the dataset for wearable-derived biomarker discovery. This design enables longitudinal characterization of disease progression and incident cardiovascular event development in a real-world clinical setting with the overarching goals of enhancing disease detection, refining risk prediction, and facilitating prevention through IRB-approved direct patient outreach. A comparator cohort without known CVD will also be enrolled to support generalizability. For all incident outcome analyses, baseline disease status will be explicitly accounted for, with only events occurring after study entry considered.

## Study aims

The central hypothesis of the HEARTBEAT study is that CVDs are associated with distinct abnormalities in biometric signals derived from smartwatch-based monitoring. These abnormalities may include, but are not limited to, alterations in HR, HR variability (HRV), oxygen saturation, as well as PPG waveforms.

The primary objectives of the study are:

1. To characterize the associations between smartwatch-derived biometric parameters and the incidence of cardiovascular events.
2. To establish biometric ‘fingerprints’ based on the wearable-derived variables that enable identification of underlying CVDs (Graphical Abstract).
3. To develop and validate machine learning models for predicting the onset of acute cardiovascular events.

The secondary objective is exploratory and aims to apply advanced AI methods to reveal both linear and nonlinear relationships between wearable-derived biometric parameters, comorbid conditions, and clinical outcomes. The findings will be utilized to develop algorithms for CVD risk stratification, patient selection, and therapeutic decision-making.

## Study design

**Figure 1:**
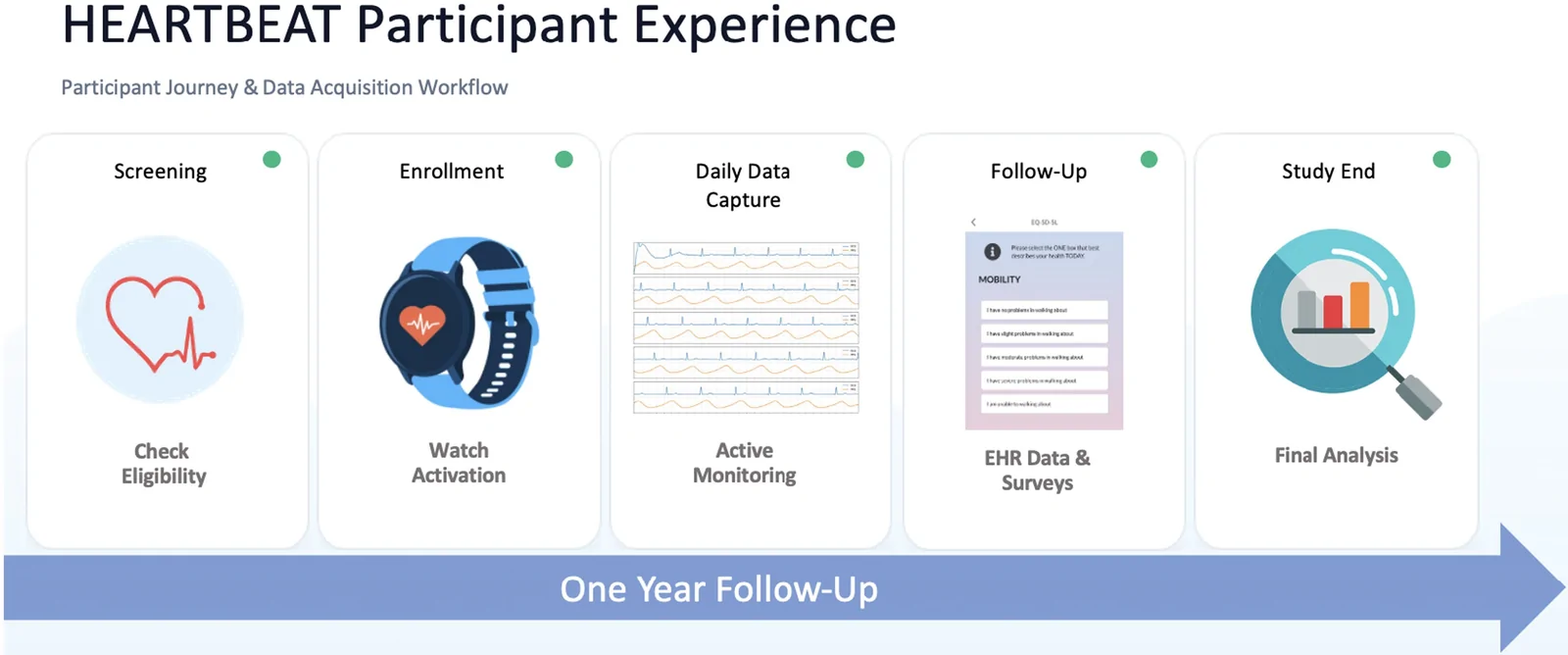
Participants’ experience in HEARTBEAT study from the pre-screening to the end of the study. Study workflow. Patients are screened for eligibility and enrolled in the study, at which point the study smartwatch is provided and activated. Continuous active monitoring then begins, with biometric data collected via the wearable device alongside periodic survey administration and extraction of relevant electronic health record electronic health record data. All data are subsequently aggregated and analysed to address the study aims.

The HEARTBEAT study is a prospective, single-arm, observational cohort study. Participants in the study will be followed up for at least 1 year using a Samsung Galaxy® smartwatch. The overall study design is depicted in *Figure 1*.

The target population includes adults with at least one diagnosed CVD as well as patients visiting a primary care provider for any reason, who will serve as a healthy control cohort. The control group will also receive the study wearable device, enabling direct comparison of biometric and outcome data against participants with established CVD. Details of inclusion and exclusion criteria are listed in *Table 1*.

**Table 1:** Inclusion and exclusion criteria for HEARTBEAT study population

Study population
**Inclusion criteria**
Patients 18 years or older.
Patients with at least one cardiovascular disease (heart failure, cardiac arrhythmias, chronic kidney disease, coronary artery disease, history of stroke/TIA, or diabetes mellitus). OR
Patients visiting a primary care provider for any reason without any of the previously mentioned cardiovascular diseases (healthy control cohort, limited up to 625 patients throughout the study).
**Exclusion criteria**
Participants who cannot read, speak, and/or understand English.
Participants with cognitive impairments who are unable to give informed consent or sign their HIPAA form.
Participants with a pacemaker.
Participants that are pregnant.
Participants with cognitive impairments affecting their ability to be compliant with wearing and maintaining wearable devices.
Participants with tattoos, scars, or skin adhesions that would not allow for PPG recordings to be collected from a wrist-worn device.
Participants with neurological disorders that may interfere with device signal quality (e.g. hand tremors).
Participants with allergies to watch and/or wristband materials.
Participants with known plans to permanently leave the state in which they were enrolled within the follow up period.
Participants who have no known medical history with any of the enrolling institutions.
Participants without a compatible smartphone (iOS 13 and Android 11 at minimum)

HEARTBEAT, Health Electronic Assessment of Risks and Trends using Biometric Equipment and Technology; PPG, photoplethysmography; TIA, transient ischemic attack.

### Ethics

The HEARTBEAT study is conducted in accordance with the Declaration of Helsinki and applicable institutional and regulatory requirements for human subjects research. The study protocol was reviewed by the Tulane University Biomedical Institutional Review Board (Protocol Number: 2024-652) and the Western Institutional Review Board-Copernicus Group (Protocol Number: 1375428). All participants provide IRB-approved informed consent prior to enrolment.

### Consent

All participants enrolled in the HEARTBEAT study provide informed consent through IRB-approved procedures before study-related data collection. Consent includes permission for collection of electronic health records (EHRs), collection of biometric data through study-issued wearable device, completion of in-application surveys, and use of de-identified data for research purposes.

### Recruitment, screening, and enrollment

Recruitment occurs in person at IRB-approved clinics and hospitals and remotely through the study website (HEARTBEATstudy.com), social media, and flyers. Enrolled participants complete a pre-screening survey reviewed by study staff to confirm eligibility. Remote recruitment will take place only through IRB-approved sites, whereas the participants already have established care in those institution.

### Monitoring and study intervention

All enrolled participants will use a Samsung Galaxy Watch for the duration of the study a Samsung Galaxy® Watch. Physiological parameters such as HR, HRV, sleep patterns, and activity level will be continuously monitored. At baseline, medical information will be obtained using standardized questionnaires via a dedicated smartphone application, along with EHR data pulls.

### Study smartwatch

Participants in the HEARTBEAT study will receive a Samsung Galaxy Watch® on loan, to be returned at study completion.

In-person participants receive device setup assistance from study staff. Remote participants receive a HEARTBEAT kit with setup instructions and study-team contact information. To ensure data standardization and protocol integrity, personal devices are not permitted. Each smartwatch is equipped with PPG, ECG, accelerometer, gyroscope, microphone, ambient light, bioimpedance, and skin temperature sensors, enabling continuous monitoring of key biometric data, as detailed in *Table 2*.

**Table 2:** List of Samsung smartwatch sensors utilized in the HEARTBEAT study and features derived from each

Sensor	Data type	Derived features
Photoplethysmography	Passive	Heart rate, heart rate variability, and O2 saturation (nocturnal only)
	Active	Oxygen saturation and blood pressure
Electrocardiography	Active	Heart rate, heart rate variability, and cardiac rhythm
Inertial measurement unit	Passive	Sleep onset/offset, sleep duration and architecture, and step count
Bioimpedance sensor	Active	Body composition
Ambient light sensor	Passive	Ambient light during sleep

The Samsung Galaxy Watch® employs a pre-validated software on sensor-captured data to generate various health metrics, including sleep patterns, HR, HRV, AF, body composition, and oxygen saturation, as detailed in *Table 2*. The watch collects data passively and through periodic participant-initiated spot-checks, balancing comprehensive data capture with user convenience.

Participants are prompted daily to perform a 30-s spot-check to record an ECG, and weekly to complete bioelectrical impedance analysis. Raw PPG data are passively collected for 1 min every 10 min while the watch is worn. Participants may initiate spot-checks at any time, allowing for additional data capture. Participants are instructed to charge their devices during daytime hours to preserve the continuity of nocturnal data collection, as sleep metrics constitute an important variable. Given a typical battery life of approximately 48 h, charging frequency is expected to average once every two days.

### Electronic medical records

Participants enrolled in the study will be continuously followed up by surveying the electronic medical records (EMRs) of the enrolling institution. At enrolment, EMR review will provide comprehensive baseline clinical data, including medical history, laboratory results, and imaging results. Throughout the follow-up period, the EMR will be continuously monitored for the occurrence of new medical conditions, clinical events, or relevant disease progression.

### Study application

Upon enrolment, participants will receive a unique access code and link to download the study mobile application (*HEARTBEAT app*), which serves as the study platform for electronic consent and scheduled tasks. To promote engagement and reflect real-world use of the technology, participants will also be able to view weekly summaries of their data, like ECG strips and HR metrics. They are informed that this constitutes their personal health information that may be shared with clinicians at their discretion, with any clinical decision-making delegated to the treating physician.

To minimize bias, no diagnoses are communicated to participants through the study platform; all reported metrics are derived from the watch's native output and are not study-generated interpretations.

In terms of compliance, it will be monitored through weekly review of participant wear-time and data completeness metrics. Participants falling below predefined compliance thresholds are contacted first via in-app notification; if adherence does not improve, a follow-up phone call is initiated to identify and address any barriers to participation. To further support sustained engagement, the study app incorporates gamification elements designed to encourage consistent device wear and timely completion of study tasks.

### Study outcomes

#### Clinical outcomes

The outcomes for the HEARTBEAT study will include cardiovascular events indicative of progression of pre-existing cardiovascular conditions, as well as the incidence of new CVDs or events. These outcomes include:

- All-cause mortality
- Cardiovascular mortality
- All-cause hospitalization
- Cardiovascular hospitalization
- Acute decompensated heart failure (ADHF) (I50.*)
- Acute myocardial infarction (AMI) (I21.*, I22.*)
- Stroke (I60.*, I61.*, I62.*, I63.*)
- Transient ischaemic attack (TIA) (G45.*)
- Cardiac arrhythmias (I44, I45, I47–I49)
- Hypertensive emergencies (I16.1)
- Revascularization procedures (CPT 92920–92945, 33510–33536)

These outcomes will be collected from the participants’ EHRs within the participant’s hospital system, which will be pulled continuously using the International Classification of Diseases Tenth Edition (ICD-10) codes. The outcomes will also be collected by extracting text from notes and reports within the EHR. This allows for adjudication of outcomes and their confirmation, as well as identification of outcomes that do not appear in the ICD-10. In addition, participants will be able to self-report these events using the HEARTBEAT mobile application.

For major clinical outcomes, including major adverse cardiovascular events (MACE) and new-onset CVDs, candidate events identified through structured EHR data or unstructured clinical documentation will undergo secondary chart review by clinically trained study investigators using prespecified clinical definitions. When uncertainty exists, source documentation, including physician notes, discharge summaries, procedure reports, imaging reports, and laboratory results, will be reviewed to confirm event occurrence and timing. Self-reported events collected through the HEARTBEAT mobile application will be recorded for research purposes and may support event identification but will not serve as the primary source for outcome adjudication.

### Patient-reported outcomes

During the study period, participants will be asked to complete surveys on patient-reported outcomes and changes in medical history via the HEARTBEAT mobile application. A set of baseline surveys will be available at enrolment, with additional surveys released periodically throughout follow-up. Surveys include:

- Medical and surgical history (every 90 days)
- Medications including analgesic medications (every 90 days)
- Sleep-related questionnaire (Pittsburgh Sleep Quality Index,^27^ every 30 days)
- Quality of life questionnaire (EQ-5D 5L,^28^ baseline and end of study)
- PRAPARE Social Determinants of Health Questionnaire^29^ (baseline and end of study)
- Illness Survey (available weekly)
- Dietary and lifestyle questions (baseline and end of study)

At the conclusion of the 1-year study period, participants will be asked to complete an end-of-study survey, which will be made available on the HEARTBEAT mobile application 1 week prior to study completion (*Figure 1*). Self-reported events are symptom-based and do not prompt chart review; records of these events are maintained by the study team for research purposes.

## Methods

### Sample size estimation

The HEARTBEAT study includes individuals with at least one of six cardiovascular or cardiometabolic conditions of interest, including HF, AF, CAD, stroke, diabetes mellitus, and chronic kidney disease, to evaluate progression of existing CVD and development of new cardiovascular events. Based on preliminary data derived from the TriNetX EHR network among individuals with these conditions, the 1-year MACE rate is estimated at 19.9% in individuals with AF and 12.3% in individuals without AF, used as a proxy for higher vs. lower AF burden. Assuming about 250 patients with baseline AF for a 10% loss to follow-up, a total sample size of 1498 individuals, including 278 with AF and 1220 without AF, yields 80% power to detect a statistically significant difference at a two-sided significance level of 0.05.

### Data analysis plan

To address the aims of the HEARTBEAT study, both traditional statistical methods and advanced machine learning techniques will be employed to analyse the collected data comprehensively.

#### Primary analysis

The primary analysis will evaluate the association between smartwatch ECG-derived AF burden and the risk of MACE during follow-up. AF burden will be quantified using daily smartwatch ECG recordings collected longitudinally throughout the study period. Specifically, participants will be instructed to acquire at least one 30-s single-lead smartwatch ECG recording per day throughout follow-up, with additional recordings allowed and encouraged. For each day, the AF burden will first be calculated as the proportion of submitted ECG recordings classified as AF. When more than one ECG recording is submitted on the same day, the daily AF burden will be calculated by averaging across all recordings from that day. AF burden over a predefined observation period will then be calculated as the average of daily AF burden values across that period.^30^

Participant-level AF burden metrics will be summarized over predefined time windows and evaluated in relation to incident MACE using time-to-event models. The composite MACE endpoint will include cardiovascular death, acute myocardial infarction, stroke or transient ischaemic attack, acute decompensated HF, and cardiovascular hospitalization.

#### Secondary analyses

Secondary analyses will evaluate additional prespecified wearable-derived biometric measures in relation to cardiovascular outcomes. These include:

1. Development and evaluation of PPG-based AF detection and estimation of PPG-derived AF burden, including its association with subsequent MACE.
2. Evaluation of longitudinal changes in wearable-derived biometric signals and their association with subsequent MACE.
3. The association between wearable-derived sleep metrics and incident MACE.

PPG-derived AF burden will be calculated using the same daily averaging framework as smartwatch ECG-derived AF burden. PPG signals will be collected passively, with monitoring performed approximately every 1 min within each 20-min interval, and will also be collected during participant-submitted ECG recordings. For each day, PPG-derived AF burden will be calculated as the proportion of analyzable PPG segments classified as AF. When multiple PPG segments are available within a day, the daily PPG-derived AF burden will first be calculated, and burden over a predefined observation period will then be defined as the average of daily PPG-derived AF burden values across that period.

#### Exploratory analyses

Exploratory analyses will include, but not be limited to:

1. Development and evaluation of multimodal PPG/ECG-based screening scores for cardiometabolic comorbidities.
2. Identification of abnormal physiological states using PPG-derived algorithms for vital sign abnormalities.
3. Development and evaluation of additional machine learning and AI models using multimodal wearable-derived signals, including algorithms for detecting or predicting additional disease states, identifying wearable-derived digital biomarkers, developing risk stratification models, and supporting treatment decisions.
4. Additional non–machine learning statistical analyses to investigate population-level associations between wearable-derived biometric measures and clinical outcomes.
5. Comparison of wearable-based prediction models with established clinical risk models (e.g. ASCVD, CHARGE-AF, CHA_2_DS_2_-VASc) to evaluate the incremental value of wearable-derived biomarkers.

#### Statistical analysis

Descriptive statistics will be generated to characterize the baseline demographics and clinical information of the study population. Baseline comparisons between participants who experience cardiovascular events and those who remain event-free will be conducted. Categorical variables will be summarized into counts and percentages and compared through Chi-square tests. Continuous variables will be represented by mean and standard deviation and assessed using the *T*-test/analysis of variance for normally distributed data or the Wilcoxon tests for non-normally distributed data. Normality will be assessed via Shapiro-Wilk tests.

Longitudinal PPG-derived features, including systolic amplitude, pulse width, perfusion index, PPG augmentation index, pulse transit time, and ΔT, will be summarized to capture baseline level, short-term dynamics, and long-term trends. Baseline levels will be described using the mean and standard deviation. Short-term variability will be quantified using the average within-subject standard deviation and the root mean square of successive differences (RMSSD), while between- and within-participant variability will be assessed using intraclass correlation coefficients. Long-term change will be estimated with linear mixed-effects models. Features will then be compared among outcome groups to identify significant differences and will also be incorporated into outcome models as time-updated exposures when appropriate, including through joint modelling frameworks or machine-learning models that integrate features across multiple predefined temporal windows. Visualization tools such as box plots and scatter plots will be utilized to illustrate distributions and patterns as needed. Time-to-event analyses will be performed using Kaplan–Meier survival curves, log-rank tests, and Cox models adjusted for relevant confounders, such as age, sex, race, baseline comorbidities, and socioeconomic factors, with the specific adjustment set tailored to the hypothesis under investigation. Clinical variables that may change during follow-up, including medication exposure and vital signs, will be incorporated as time-varying covariates when available. Biomarkers will be screened through univariable analyses in terms of their associations with cardiovascular events and subsequently confirmed by multivariable models, with interaction effects explored for significant biomarkers and comorbidities.

Statistical analyses will be conducted using R software (version 4.2.0), and a two-sided significance level of 0.05 will be considered statistically significant. The primary analysis will be interpreted at a two-sided significance level of 0.05. Secondary analyses will be adjusted for multiple comparisons where appropriate using false discovery rate control, while exploratory analyses will be interpreted as hypothesis-generating without formal claims of statistical significance.

#### Machine learning analysis

In addition to traditional statistical methods, exploratory machine learning models to predict CVDs and events using biometric data obtained from smartwatches and smartphones will be developed and validated. These include patterns in HR, HRV, SpO_2_, ECG, and PPG waveforms. These signals will be quantified longitudinally and their relationship to clinical events will be tested, to delineate biometric ‘fingerprints’ that discriminate latent or progressing cardiovascular pathology.

To achieve this objective, predictive models that integrate continuous smartwatch data with electronic health record variables will be constructed and validated. Machine-learning methods will be employed to identify non-linear relationships linked to outcomes such as AF, CAD, and HF hospitalization. Model development will provide clinically interpretable markers that facilitate earlier disease detection and personalized cardiovascular risk stratification.

Missing smartwatch data, primarily arising from periods of device non-wear, will not be imputed. Instead, analyses will be based on available high-quality recordings after standard preprocessing steps to remove low-quality or incomplete wearable signal segments.

Multiple machine-learning models will address distinct analytic objectives rather than a single unified model. Each analysis defined in the primary and secondary analysis sections will involve dedicated statistical or machine learning models tailored to the specific objective. Inputs will include longitudinal wearable data and clinical variables such as vital signs, medication exposure, temporal features, biometric measurements. Feature type will depend on the objective; for example, PPG-derived AF burden will utilize raw, high-resolution PPG signals, whereas biometric change analyses will use derived features summarized over prespecified windows (e.g. daily summary). Relevant clinical and demographic covariates will be included.

The machine learning models referenced in this study (e.g. AF detection and screening algorithms) are developed within the broader HEARTBEAT study, but not solely within this cohort. Rather, they are developed and validated using larger independent datasets in addition to this cohort, consistent with standard practice.

Depending on the analytic objective, candidate model classes may include traditional machine-learning approaches, such as gradient boosting machines, random forests, and elastic net models. For high-dimensional or complex longitudinal wearable signals, deep learning architectures for signal processing, including ResNet-, EfficientNet-, or transformer-based models, may be evaluated. For dynamic risk prediction, longitudinal modelling approaches, including joint models and other temporal prediction frameworks, may also be considered. Model performance will be assessed using appropriate validation strategies, including cross-validation or held-out testing where applicable, and task-specific metrics such as AUROC, sensitivity, specificity, positive predictive value, calibration, and time-dependent discrimination.

In this cohort, HEARTBEAT primarily uses features derived from these algorithms for hypothesis-driven analyses. Model development is performed separately and does not contribute to hypothesis testing or alpha expenditure within the present study. Clinical outcomes, including disease onset and hospitalization, are ascertained independently and are not used for model training; instead, training relies on signals such as ECG-confirmed AF events and physiologic measurements. This design maintains a clear separation between model development and outcome evaluation, reducing the risk of data leakage.

### Study launch and enrollment

The HEARTBEAT Study was launched on 8 October 2024, at which point the first participant was enrolled and the first watch activated. After evaluation of study operational and quality metrics, study enrolment has proceeded without any further restriction. Based on operational metrics, there were 897 enrolled participants as of 15 December 2025.

## Discussion

The HEARTBEAT study is designed to shift cardiovascular care from a clinic-bound, episodic model into a model of continuous, patient-individualized care. By collecting continuous multimodal smartwatch signals from a large cohort of adult participants with diverse CVD conditions, the study aims to build a comprehensive atlas of ‘biometric fingerprints’. Foundational models trained on PPG, HR, HRV, SpO_2_, bioimpedance, and activity data may potentially identify physiologic tipping points well before symptoms emerge, effectively transforming each smartwatch into an early-warning system.

Continuous physiologic data will allow predictive models to track CVD trajectories in real-time, identifying early signs of rhythm instability, ischaemic, or fluid overload before symptom development, enabling personalized treatment and monitoring strategies.^31^ This represents a shift from reactive, visit-based care to proactive, individualized management that is matched to each patient’s biologic profile. However, because participants have access to selected wearable outputs, such as ECG and HR data, and share them with clinicians, behavioural changes, additional clinical evaluations, or treatment modifications may occur during follow-up. Observed associations may therefore partly reflect the real-world influence of wearable-guided clinical decision-making rather than passive physiologic monitoring alone.

Importantly, the study cohort has been intentionally designed to capture broad representation across comorbidities, race, ethnicity, and socioeconomic status. This diversity ensures that resulting algorithms are validated in real-world complexity rather than homogeneous trial populations, an essential step toward building equitable AI tools that perform consistently across demographic groups and resource settings. By integrating participants’ full longitudinal medical history from the EMR, including diagnoses, procedures, medications, labs, and encounters, the HEARTBEAT study grounds every prediction in the rich clinical narrative that truly shapes patient care and health-system decision-making.

Although the HEARTBEAT study utilizes Samsung wearable devices, the physiologic signals analysed, such as HR, HRV, and oxygen saturation, are measured using similar sensing principles across many modern wearable platforms. Consequently, the biological patterns and digital biomarkers identified in this study may generalize to other wearable ecosystems. However, differences in device hardware affect the direct application of algorithms trained on raw data, such as PPG-based AF detection models, which will require further cross-platform calibration and validation.

## Data security and privacy

Several measures have been implemented to protect the participant’s data and privacy throughout the study. The EHRs, including demographics, vitals, labs, diagnosis, and medication history, will be de-identified and stored in an ISO 27001-certified data capture system. De-identified study data will be deleted within six months after the last participant data collection is completed.

The data collected from Samsung Galaxy Watch will be initially stored and processed on Samsung’s HIPAA-compliant cloud backend. Any data collected through the study app or smartwatch are de-identified and cannot be used to identify participants. No one at Samsung will have access to a participant’s personal information. Every smartwatch will be under the management of Samsung’s Knox protocol, which is SOC2 and DISA compliant.

## Summary

The HEARTBEAT study is a prospective, single-arm, decentralized observational clinical study designed to test the hypothesis that CVDs are associated with distinct patterns of biometric smartwatch-derived signals. By integrating continuous, real-time data from consumer-grade wearable devices with longitudinal clinical outcomes, the study seeks to identify novel digital biomarkers and predictive signatures of CVD. In doing so, HEARTBEAT is poised to make a significant contribution to the future of cardiovascular care, advancing a more digital, personalized, and proactive model of medicine.

## Data governance, ethics, and reporting standards

The HEARTBEAT study was approved by the institutional review board, and all participants provide informed consent before enrolment. The study is designed and reported in accordance with STROBE guidelines and aligned with the minimum standards of the CODE-EHR framework for research using linked healthcare data. Wearable-derived biometric data are linked to institutional EHR records under IRB-approved procedures, with prespecified cohort definitions, follow-up windows, outcome definitions, and analytic plans. Clinical variables and outcomes are extracted using standardized coding systems, including ICD-10, CPT, LOINC, and RxNorm, with major clinical outcomes further reviewed by clinically trained investigators using prespecified definitions. De-identified data are stored within secure HIPAA-compliant infrastructure, and code lists, extraction logic, and analytic code will be made available upon reasonable request following publication of the primary results.

## Supplementary Material

ztag132_Supplementary_Data

## Data Availability

This study design manuscript does not report participant-level analytical results. The individual participant datasets generated during the HEARTBEAT study, including wearable-derived biometric signals, derived features, survey responses, and linked EHR outcomes, are not publicly available because of participant privacy protections, institutional policies, IRB requirements, and applicable data-use agreements. De-identified data may be considered upon reasonable request to the corresponding author after completion of the study and publication of the primary results. Any access to study material, including protocol or statistical plan, may be considered by the corresponding author upon reasonable request.

## Lead author Biography

**Nassir Marrouche, MD**, is Professor of Medicine and Director of the Tulane Heart & Vascular Institute at Tulane University School of Medicine, New Orleans, USA. A globally recognized cardiac electrophysiologist, he has led landmark multicenter trials that have shaped contemporary AF management, including CASTLE-AF, CASTLE-HTx, and DECAAF II. His research spans AF ablation, cardiac imaging, and digital health, with findings published productively in top-tier journals including the *New England Journal of Medicine*, *Circulation*, and *JAMA*.

## Supplementary material

Detailed questionnaires assessing participants' dietary habits, intercurrent illnesses during the study period, such as colds, and medication use are provided in the Supplementary Materials (Supplementary), available at *European Heart Journal - Digital Health*.
