# Supplementary material for: Health Electronic Assessment of Risks and Trends using Biometric Equipment and Technology (HEARTBEAT): study design and rationale
Source: Eur Heart J Digit Health. 2026 Aug 21;7(7):ztag132. doi: 10.1093/ehjdh/ztag132 (PMC13528244; doi:10.1093/ehjdh/ztag132)
Supplement: Supplementary file 1 [file ztag132_supplementary_data.zip › Dietary Habits Survey.docx]

**Dietary Habits Survey**

1. **How would you rate your overall habits of eating healthy foods?**
   - Very Poor
   - Poor
   - Fair
   - Good
   - Excellent
2. **How often do you eat fast food, fried food, or packaged snacks high in fat, salt, or sugar?**
   - More than 5 times a day
   - 4-5 times a day
   - 2-3 times a day
   - Once a day
   - Less than once a day
3. **How many meals do you consume in a day? (Consider every fruit, every yogurt, or a glass of milk as a separate meal.)**
   - 1-2 meals a day
   - 3-4 meals a day
   - 5-6 meals a day
   - More than 6 meals a day
4. **Do you eat fresh fruit and/or vegetables every day?**
   - Yes
   - No
5. **Do you drink at least 6 glasses of water every day?**
   - Yes
   - No
6. **When eating at home, your meals are usually:**
   - Completely homemade
   - Partly homemade
   - Reheated frozen food
   - Take-away meals
7. **How often do you cook lunch or dinner at home?**
   - Everyday
   - Three times a week
   - Twice a week
   - Once a week
   - Once every two weeks
   - Once a month
   - Never
8. **Have you been advised to follow any dietary restrictions? (Please check all that apply)**
   - Low calorie
   - Low cholesterol
   - Low salt/sodium
   - Low protein
   - Low fat
   - High fiber
   - Other (please specify: ______)
9. **Your eating schedule is typically:**
   - At fixed times
   - Only when you are hungry
   - At any time of the day
10. **How often do you eat during the night (from 00:00 to 06:00 AM)?**
    - At least three times a week
    - Only on weekends
    - Once a week
    - Rarely
    - Never
11. **Do you have any eating or digestion problems such as (Please check all that apply):**
    - Chewing
    - Diarrhea
    - Gas
    - Swallowing
    - Indigestion
    - Constipation
12. **What best describes your usual diet?**
    - Vegan - Plant-based foods only
    - Vegetarian - Primarily plant-based foods, but includes dairy
    - Omnivore - An assortment of meat, seafood, vegetables, dairy, and grains
    - Carnivore - Meat, seafood, and dairy food several times a week
    - Top of food chain - Meat, seafood, or dairy at almost every meal
13. **Where do you primarily buy your food?**
    - Directly from farms, markets, gardens, and other local fresh food producers
    - Organic food markets
    - Supermarkets for some items, organic food stores for others
    - Convenience stores and prepared food from restaurants
    - Fast food and take-away
14. **Has your weight changed in the past year?**
    - No change
    - Gained weight
    - Lost weight
15. **Do you exercise for at least 30 minutes, three times a week?**
    - Yes
    - No
