# Supplementary material for: Health Electronic Assessment of Risks and Trends using Biometric Equipment and Technology (HEARTBEAT): study design and rationale
Source: Eur Heart J Digit Health. 2026 Aug 21;7(7):ztag132. doi: 10.1093/ehjdh/ztag132 (PMC13528244; doi:10.1093/ehjdh/ztag132)
Supplement: Supplementary file 1 [file ztag132_supplementary_data.zip › Study Illness Survey.docx]

Q1. Have you had symptoms of cold/flu/covid-19 in the past week?

Response choices: Yes, No

Format: radio buttons

- If yes, approximately when did your cold/flu/covid-19 start?

Response choices: 1, 2, 3, 4, 5, 6, 7, >7, Not sure

Format: dropdown list (or calendar)

- If yes, was your cold/flu/covid-19 accompanied by a fever?

Response choices: Yes, No, Not sure

Format: radio buttons

- If yes, are you still experiencing symptoms?

          Response choices: Yes, No, Not sure

Format: radio buttons

References for standard flu/cold questionnaires on which this survey was based are available here:

- [Flu-PRO (37 items)](https://bmcinfectdis.biomedcentral.com/articles/10.1186/s12879-015-1330-0)
- [Wisconsin Upper Respiratory Symptoms Survey (WURSS) (11 items)](https://www.fammed.wisc.edu/files/webfm-uploads/documents/research/wurss-11.pdf)
- [Wisconsin Upper Respiratory Symptoms Survey (WURSS) (21 items)](https://www.fammed.wisc.edu/files/webfm-uploads/documents/research/wurss-21.pdf)
- [Wisconsin Upper Respiratory Symptoms Survey (WURSS) (24 items)](https://www.fammed.wisc.edu/files/webfm-uploads/documents/research/wurss-24.pdf)
